# Ethical Considerations on Artificial Intelligence, Health and Health Care. All That Glisters Is Not Gold

**DOI:** 10.1111/eci.70254

**Published:** 2026-09-02

**Authors:** Piero Portincasa, Mohamad Khalil, Pierfrancesco Novielli, Sabina Tangaro

**Affiliations:** ^1^ Clinica Medica “A. Murri”, Dipartimento di Medicina di Precisione e Rigenerativa e Area Jonica‐(DiMePRe‐J) Università degli Studi di Bari Aldo Moro Bari Italy; ^2^ Dipartimento di Scienze del Suolo, della Pianta e degli Alimenti Università degli Studi di Bari Aldo Moro Bari Italy; ^3^ Istituto Nazionale di Fisica Nucleare, Sezione di Bari Bari Italy

**Keywords:** clinical medicine, ethics, explainable AI, generative AI, interpretable machine learning, network medicine

## Abstract

**Background:**

Artificial intelligence (AI) is transforming global health care through innovations in deep learning, generative models and agentic AI systems. Traditional reductionist approaches to complex pathophysiological pathways fail to capture the true complexity of disease, motivating the adoption of network medicine (NM), which models biological systems as dynamic, interconnected networks. When combined with AI, NM enables integration of multiomic data and better characterizes disease mechanisms to guide precision therapies. Nevertheless, the rapid diffusion of AI in health care also raises profound ethical, regulatory and social challenges, since only a small fraction of AI tools have achieved routine clinical use, often due to limited generalizability, opaque algorithms and workflow incompatibility.

**Methods:**

Key strategies in this scenario are explainable AI (XAI) and counterfactual reasoning, which enhance transparency, accountability and fairness. These methods allow clinicians and regulators to interpret model decisions, identify biases and ensure human oversight in high‐stakes contexts. Ethical frameworks such as the European Commission's Assessment List for Trustworthy AI (ALTAI) operationalize these goals through auditable requirements spanning transparency, fairness, privacy and societal well‐being.

**Results:**

Yet, challenges persist, including algorithmic bias, data inequity and variable regulatory standards across regions. Machine learning and generative AI hold significant promise for improving diagnostics, drug discovery and population health, but their deployment must be guided by fairness, transparency and human rights principles. Indeed, inadequate governance risks can impact the already existing health inequalities.

**Conclusions:**

For these reasons, the convergence of AI, NM and telemedicine requires a co‐evolutionary model which must be rooted in ethical design, rigorous validation and equitable global implementation. Only by aligning technical innovation with sound ethical frameworks and explainability standards will AI become a highly transformative yet trustworthy force in the field of precision and public health.

## Graphical abstract

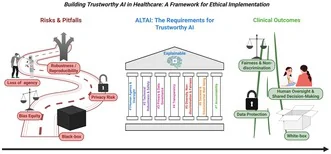

## 1 Introduction

Since artificial intelligence (AI) and modern computing emerged almost simultaneously in the 1950s, medicine has consistently sought to apply computational methods to clinical problems, from early rule‐based diagnostic systems to today's deep learning, generative AI and agentic AI applications [1].

This implementation is changing health and health care delivery globally and rapidly [2, 3] since the traditional approach based on the reductionist interpretation of human bio‐pathophysiology will not catch the true nature of complex diseases. A more holistic approach is the network medicine (NM) which provides a natural description of the complex interplay of diverse components within a system, including biological systems [4], as well as network medicine to address the complexities of disease and improve therapeutic outcomes [5]. A further step implies NM combined with AI to better unravel the manifestations of complex diseases and developing precise therapies by integrating multiomic data and leveraging advanced computational techniques. In this respect, NM and AI besides being complementary, become coevolutionary [6]. In economic terms, the AI healthcare market is projected to grow at a compound annual growth rate (CAGR) of 37.5% through 2030, reaching an estimated value of US$208.2 billion [7].

The anticipated huge potential benefits from AI in health care, however, must be weighed up against several high risks, since AI will inevitably affect several aspects related to technologies and tools, diagnostics, early disease detection, therapeutic protocol development, drug discovery, personalized treatment strategies, patient care and support [7]. In this scenario, human values and goals will ultimately be affected as well [8], depending on significant ethical challenges that must be addressed to ensure equitable and effective patient care [9]. This aspect is essential since the implementation of AI and biomedical applications must take into account the prevention of algorithmic bias, protection of patient privacy and regulation of novel technologies as they move from the research settings into practical, real‐world scenarios.

Although AI systems have consistently demonstrated promising results across numerous retrospective medical studies, only a few have successfully transitioned into everyday clinical use [10]. The process can require time to enter everyday practice. In a relatively simple model, the AI‐based electronic stethoscope shows promise as a reliable and accessible tool for atrial fibrillation screening. Potential applications in primary healthcare and general population screening are anticipated with extension to cardiovascular medicine and primary healthcare [11].

## 2 From AI Development to Clinical Implementation

Critics have noted that the real‐world effectiveness of AI tools may be lower than suggested by retrospective analyses, as such systems can prove too complex, slow or impractical for clinical workflows and unanticipated challenges may arise from human–AI interactions [10, 12].

This is a call for sound ethical design, rigorous validation, and equitable worldwide deployment of newer AI tools [13] increasingly involved in health care aspects of biomedical discovery, social aspects, communication and interaction and information management and distribution [2]. However, this transition from algorithmic promise to clinical utility begins before model training. If the underlying data are incomplete, biased, poorly measured or non‐representative of the intended population, downstream validation and post hoc explainability cannot fully repair the resulting harms. Data quality is therefore not merely a technical prerequisite for performance, but a core ethical condition for safe and fair clinical implementation.

### 2.1 Data Quality and EDA as Ethical Safeguards

The principle known since early computing as ‘garbage in, garbage out’ applies to clinical AI in a particularly consequential way: no algorithm, however sophisticated, can recover information that was never present in the training data [14, 15]. Exploratory data analysis (EDA), the systematic inspection of a dataset before model fitting, should therefore be regarded not as a preliminary technical step, but as a first‐line ethical safeguard. It is the stage at which sample size, representativeness, class distribution, missingness, feature structure, outliers, data provenance and possible sources of confounding can still be examined and, where feasible, corrected before they are encoded into model behaviour and translated into clinical decisions. The ethical role of EDA lies in making visible the conditions under which a model can or cannot be trusted. Data quality issues can be organized into three related families. At the level of the dataset itself, an insufficient sample size limits statistical power and generalizability; non‐representative sampling means that the population later exposed to the model differs from the population it was trained on, a mismatch that has been shown to translate directly into biased clinical predictions [16]; class imbalance, common in rare‐disease or adverse‐event prediction, can inflate apparent accuracy while masking poor performance on the minority class, which is often the one of greatest clinical interest. At the level of the features describing the data, irrelevant variables introduce noise and high‐dimensional feature spaces, typical of omics, radiomics and electronic health record data, require principled feature selection rather than exhaustive inclusion. At the level of the model itself, overfitting produces a system that reproduces the idiosyncrasies of its training set rather than a generalizable clinical signal, while underfitting fails to capture even the structure that is present. Each of these issues carries an ethical cost, not only a statistical one: together they determine whose data a model has learned from, and therefore whose outcomes it can be trusted to predict.

A further constraint operates upstream of any technical analysis: data ownership and access. Clinical and multi‐omic datasets are rarely the unrestricted property of the investigator analysing them; their use is bound by consent scope, institutional data‐sharing agreements and, increasingly, national or supranational data‐protection law. Which data can be used, and for which purpose, is itself an ethical determination that precedes and constrains the exploratory analysis described above. These same data‐quality deficits resurface downstream, once models move toward clinical validation, as discussed in the following section.

### 2.2 Clinical Validation and Regulatory Pathways

Once such data‐related risks have been identified and mitigated, an equally significant question emerges: who determines whether a model is ready for clinical use, and how?

Several potential legal and ethical challenges arise in this respect, including aspects related to human rights [17] and, when addressing human health issues, there is an active debate regarding which tools require evaluation (e.g., from Food and Drug Administration (FDA) in US), how evaluations should be conducted and who is responsible for health consequences (e.g., the developer?) [2, 18, 19].

Some AI tools have now progressed beyond pilot testing and achieved regulatory and administrative endorsement. The U.S. FDA has been authorizing AI and machine learning products at an increasing rate, primarily through regulatory clearances rather than full approvals [20]. While these clearances mark an important step toward clinical integration, they often rely on retrospective, single‐institution datasets that are proprietary and unpublished. To strengthen confidence in AI‐assisted medicine, the field must adopt higher standards for transparency, validation and the demonstration of tangible benefits on patient outcomes [10].

The use of AI has been debated within the EU pointing to the general rule that people and businesses can ‘enjoy the benefits of AI while feeling safe and protected’ by appropriate regulations [21, 22]. The policy should ensure equitable outcomes with respect for democratic values, human rights and the rule of law frameworks for responsible innovation [7]. Transparency of methodologies and appropriate informed consent for the patients, use of professional standards for AI stakeholders (which include developers and rightsholders of AI, investors, downstream providers and people who market it, and end users including non‐clinician researchers) and safety of AI products to prevent harm, have been partly underscored in The European Commission AI Liability Directive [23]. Additional issues to be considered include the risk of bias corrupting specific AI algorithms and the rise of health inequalities due to the use of effective AI only in a few protected demographic groups [24]. Indeed, locally tailored AI solutions can ensure equitable and sustainable educational opportunities in resource‐limited settings [25]. The risk of ineffectively governing bias in ‘black box’ containing decision‐making algorithms must also be taken into account as well [26]. Lastly, the protection of human privacy needs to reach safe standards, since the processing of personal data is in the hands of AI. This aspect has already been addressed by the European Convention on Human Rights and Biomedicine [7].

Beyond these region‐specific frameworks, the US and EU approaches differ not only in detail but in underlying logic. FDA oversight of AI‐based medical devices operates within a product‐safety paradigm inherited from traditional medical device law: a specific tool is evaluated for safety and efficacy through a risk‐tiered premarket pathway (510(k), De Novo, or PMA), historically under the assumption that, once cleared, a device remains unchanged. Because this assumption does not hold for AI/ML systems capable of learning from new data, the FDA has recently introduced the Predetermined Change Control Plan (PCCP), which allows manufacturers to pre‐specify the scope of future algorithm updates and have them reviewed once, rather than resubmitting for each change [27]. The EU AI Act, by contrast, does not regulate AI as a category of medical device but as a horizontal risk class: systems used for diagnosis, triage or clinical decision support are classified as high‐risk based on their potential impact on health, safety or fundamental rights and are subject to layered obligations, data governance, technical documentation, human oversight, transparency, that apply on top of, rather than instead of, existing medical device regulation. This distinction has practical consequences: a device cleared by the FDA is not thereby compliant with EU AI Act obligations, and a CE‐marked device still requires a separate US regulatory pathway, so that ethical and technical requirements developed for one jurisdiction cannot simply be transposed to the other. This divergence is likely to persist as other regions develop their own frameworks, often drawing selectively from both models.

## 3 Ethical Challenges Across AI Paradigms and Model Families

### 3.1 Discriminative Versus Generative AI

The ethical challenges reviewed so far, data quality, regulatory evaluation, largely concern discriminative models: systems trained to predict a label, risk score or classification from patient data, where the central concerns are accuracy, bias and the faithfulness of post hoc explanations to an existing decision boundary. Generative AI, which produces new content, text, images or synthetic patient data, rather than a discrete prediction, raises a partly distinct set of ethical questions. Because a generative model's output is not a single verifiable decision but content that can closely resemble authentic clinical documentation or communication, concerns shift toward hallucination (plausible but unsupported content), disclosure (whether patients and clinicians are informed that content is AI‐generated), and the boundary between assistance and unsupervised authorship of clinical text.

It is worth noting that not all foundation models are generative in this sense: models such as SleepFM are pretrained on unlabeled physiological data through self‐supervised contrastive learning, yet the resulting representations are subsequently fine‐tuned for discriminative tasks, disease risk prediction from polysomnography, rather than content generation [28]. This illustrates that the discriminative/generative distinction depends on how a model's output is used, not merely on its training paradigm.

Further efforts are devoted to promoting systematic ethical assessments in GenAI research, addressing the current inadequacies in ethical discussions and guidelines for its applications in health care. Guidelines are being developed in this field when dealing with issues related to public health applications in health care. In particular, GenAI is a powerful technology and can play a role in health care and beyond. Nevertheless, poor ethical standards in these roles can create inefficient administrative services while worsening health outcomes, and this aspect is essential for clinicians, patients and the general public. Developing appropriate recommendations will pave the way to further applications to general AI and facilitate more responsible and trustworthy development of technology [29]. The importance of disclosure also needs to be considered. In a recent survey, participants preferred AI‐drafted messages but reported slightly lower satisfaction when informed that AI was involved, highlighting the need for ethical disclosure practices that empower patients [30].

### 3.2 Supervised, Unsupervised, Deep Learning and Interpretable Models

Ethical implications of transparency and bias also vary with the learning paradigm and the model family employed. Supervised learning, the paradigm underlying most of the discriminative models discussed above, maps labelled patient data to a known outcome, so that trustworthiness can, in principle, be checked against ground‐truth labels: bias appears as a systematic discrepancy between predicted and true outcomes across patient subgroups. Unsupervised learning is used instead when the outcome itself is unknown or ill‐defined, when dimensionality is high, as in omics, radiomic or electronic health record data, or when interactions among variables are complex and non‐linear, as in multi‐omic and multimodal datasets. Typical clinical questions here are exploratory rather than confirmatory: are there distinct patient subgroups within a disease, and do these subgroups associate with prognosis or treatment response? Because there is no ground truth against which to validate a discovered subgroup or cluster, bias and instability are harder to detect and require different safeguards, such as stability analysis across resampling and independent clinical validation of any subgroup structure before it informs care.

Ethical scrutiny must also be calibrated to the model family. Tree‐based methods (e.g., random forests, gradient boosting) may provide accessible summaries of model behaviour, such as feature‐importance measures, making transparency and bias auditing comparatively more direct than in many deep learning models. However, such summaries are not causal explanations and may themselves be unstable or biased by feature correlation and measurement structure. Deep learning models, by contrast, rarely offer this native interpretability: their transparency depends entirely on external, post hoc methods, several of which are described below, making the choice between an intrinsically interpretable model and a more accurate but opaque one itself an ethical decision, not merely a technical one.

Black‐box predictors pose particular risks in high‐stakes decisions, where responsibility ultimately rests with clinicians. For such settings, it has been argued that intrinsically interpretable models should be the default, rather than retrofitting post hoc explanations onto opaque systems [31].

### 3.3 Retrieval‐Augmented and Agentic AI

Retrieval‐augmented generation (RAG) is increasingly proposed as a way to improve the clinical reliability of generative AI. In a typical RAG pipeline, external medical knowledge, such as clinical guidelines, biomedical literature, electronic health records or curated knowledge bases, is indexed, retrieved in response to a user query and then provided to the generative model to support the final answer. This architecture may reduce reliance on static pretraining knowledge, improve access to up‐to‐date evidence and support more contextualized and personalized responses in health care [32].

However, RAG should be regarded as a hallucination‐mitigation strategy, not as a hallucination‐elimination strategy. Its performance depends on the quality of the retrieved sources, the representativeness of the corpus, the ranking procedure and the model's ability to integrate evidence faithfully. Retrieval may introduce errors when irrelevant, outdated, biased or contextually inappropriate sources are selected. Recent biomedical RAG work has shown that medical question answering can be affected by irrelevant context, poorly targeted queries and retriever bias toward dominant corpora, motivating approaches such as rationale‐guided retrieval, filtering of distracting snippets, balanced retrieval across biomedical sources and reranking [33].

A further ethical risk is apparent transparency. The presence of citations or retrieved sources can make a generated answer appear evidence‐based, but source grounding alone does not guarantee safe clinical communication. A response may be literally accurate but still misleading if it decontextualizes evidence, omits relevant countervailing information, reinforces a patient's presupposition or fails to communicate uncertainty, base rates and risk–benefit trade‐offs. Therefore, RAG systems should be evaluated not only for factual correctness and citation accuracy, but also for source quality, contextual fidelity, communicative adequacy and likely interpretation by clinicians or patients [34].

These concerns become even more consequential in agentic AI systems, where the model does not merely answer a question but plans steps, selects tools, retrieves information, executes workflows or initiates actions within a digital environment. In health care, such systems could support triage, documentation, scheduling, follow‐up, medication reconciliation or care coordination. However, when an AI system moves from response generation to action, the risks of inappropriate delegation, opaque intermediate reasoning, automation bias and loss of accountability increase. Agentic AI therefore requires explicit boundaries of autonomy, human authorization for clinically consequential actions, logging of intermediate steps, traceability of retrieved sources and tool use, fail‐safe mechanisms and post‐deployment monitoring. In this sense, RAG and agentic AI can improve clinical usefulness only if embedded within governance structures that preserve human oversight, auditability and accountability [35].

## 4 Explainability, Counterfactual Reasoning and Human Oversight

### 4.1 Frameworks and Methods for Explainability

The rising debate over AI versus human judgement has become a focal point in discussions about the future of medical practice. Recent work has shifted from comparing AI and clinicians toward human‐in‐the‐loop models, where AI supports clinical decision‐making [36, 37]. Studies show that AI–human collaboration often improves diagnostic accuracy, as seen in radiology and other specialities, though results depend on task complexity and clinician experience. Less experienced practitioners appear to benefit most from AI guidance [38, 39, 40].

However, AI assistance can also introduce challenges, such as reduced specificity, user overreliance, or misjudgement [12]. The presentation format of AI outputs, probabilities, text, or annotated images can influence clinical interpretation [41]. These findings emphasize that effective and ethical integration of AI requires transparency, rigorous validation, thoughtful interface design and sustained human oversight to preserve accountability and patient safety.

Human oversight is only effective if clinicians can make sense of how an AI system arrives at its output. This need for intelligibility has motivated the development of explainable AI (XAI), a set of methods that render model behaviour interpretable to end users.

A pragmatic view of autonomy in medicine mirrors the graded autonomy of self‐driving systems: full automation without meaningful human backup is neither realistic nor desirable. As Topol argues, the goal is synergy—clinicians remain responsible for monitoring, understanding and, when needed, overruling algorithmic outputs. In this co‐driving paradigm, explainability functions as the dashboard and guardrails that make human oversight possible [42].

Recent theoretical work has emphasized that explainability should not be viewed as a single property but as a multidimensional construct underpinning trust in medical AI. Ali et al. [43] proposed a unifying framework for trustworthy and explainable AI in clinical decision‐making, encompassing four complementary dimensions: data transparency, model transparency, post hoc explainability and explanation assessment. This layered structure reinforces that trustworthiness depends not only on the algorithm itself but on the entire explanatory process—from dataset documentation and bias analysis to the evaluation of explanation quality and clinical usability. Within this perspective, explainability operates as a proactive safety mechanism, allowing clinicians and regulators to detect dataset shift, unintended correlations, or model drift before these cause harm.

Importantly, Ali and colleagues argue that technical interpretability alone is insufficient for building trust: explanations must be causally coherent, human‐understandable and actionable within established care pathways. Their framework thus operationalizes the ethical principle of transparency, linking it directly to accountability and patient safety across the AI life cycle.

Where deep learning is indispensable—for example, in medical imaging—XAI methods can still make model behaviour inspectable [44, 45]. Gradient‐based visualizations such as Grad‐CAM highlight image regions supporting the prediction [46, 47]; model‐agnostic tools like LIME and SHAP provide local or global feature attributions for tabular and multimodal data [48]; and inspection of attention patterns in transformer architectures can assist sense‐making for sequence tasks [49]. Each serves distinct clinical needs: a radiologist may benefit from spatial heatmaps, whereas a prognostic model in internal medicine often requires patient‐specific feature rankings. These tools, however, should be treated as decision aids, not ground truth: attention weights are not explanations per se, and saliency or attribution maps can be unstable if not validated.

To address this evaluation gap, clinician‐informed checklists have recently been proposed to standardize how explanations are assessed and reported, spanning purpose, clinical attributes (domain relevance, coherence, actionability), decision attributes (correctness, confidence, consistency, causal validity) and model attributes (bias and fairness, robustness) [50]. Such frameworks move XAI evaluation beyond demonstrating that a method runs, toward evidence that its explanations are clinically relevant, stable across repeated queries and free of the disagreement between methods that different XAI techniques applied to the same model have been shown to produce.

### 4.2 Counterfactual Reasoning for Fairness and Accountability

Beyond post hoc attribution, counterfactual explanations answer the clinician's natural ‘what if?’ the minimal, clinically plausible changes to a patient's features that would alter the model's output. Counterfactuals are useful for triage, treatment eligibility and appeal/review because they translate model behaviour into actionable levers. To be safe and meaningful, they must respect domain constraints (physiologic plausibility, monotonic relations where appropriate, preservation of legitimate correlations) and be evaluated for proximity, sparsity, feasibility and fidelity to the original model.

Recent work indicates that generative approaches can produce counterfactuals that remain close to the data manifold. In computer vision, diffusion‐based methods guide a denoising diffusion process with the target classifier to generate counterfactual images that are realistic and diverse while avoiding adversarial‐type artefacts; in addition, they introduce evaluation tools to detect spurious correlations beyond standard fidelity and sparsity checks. Although developed on benchmark image datasets, these techniques are directly relevant to clinical imaging pipelines, where plausibility, diversity and auditability of counterfactuals are critical for downstream review, safety oversight and documentation within model governance [51].

Beyond image and tabular generation, counterfactual reasoning offers a proactive framework for assessing fairness and accountability in medical AI. It allows one to ask whether a system's recommendation would change if a patient's sensitive attributes—such as sex, race or socioeconomic background—were altered while all other clinically relevant characteristics remained constant. This can reveal unfair dependencies and hidden proxies for protected attributes that may remain invisible in aggregate performance metrics. Counterfactual analysis therefore operationalizes fairness as a measurable property rather than a general ethical aspiration: it moves beyond simple correlation by examining the putative causal influence of demographic variables on predictions and allows developers and regulators to test whether decision boundaries reflect impermissible dependencies on variables that should be ethically or legally irrelevant to care delivery. In regulatory contexts, counterfactual analysis provides an auditable evidence trail by documenting how models behave under hypothetical but clinically plausible variations of patient data, thereby informing subgroup‐specific evaluation and bias mitigation across the total product life cycle. This approach aligns with emerging regulatory frameworks, including the EU Artificial Intelligence Act and recent FDA guidance, both of which emphasize transparency, traceability and explainability in high‐risk medical AI. Counterfactual reasoning thus complements existing fairness audits by linking algorithmic accountability directly to causal analysis and ethical oversight across the entire product life cycle [52].

## 5 Trustworthy Governance in Public‐Health AI: Telemedicine and ALTAI

### 5.1 Public Health Principles and Telemedicine as Case Study

A JAMA summit report on AI called for an ecosystem capable of generating rapid, efficient, and generalizable knowledge about the health consequences of these tools [2]. More broadly, more than 80 AI ethics frameworks have been developed globally, yet none has been specifically designed to address the role of AI and telemedicine in public health [53]. Extending this analysis to frameworks developed in AI ethics, bioethics, medical ethics and public health ethics points to a consistent set of pillars: beneficence, nonmaleficence, autonomy and justice, also endorsed by the WHO [53, 54] (Table 1).

**TABLE 1:** General ethical principles in AI‐based telemedicine.

Ethical principles for AI and telemedicine for public health	Issues from AI ethics [54, 55, 56]	Issues from bioethics, medical ethics, telehealth, and telemedicine [57, 58, 59, 60]	Issues from public health ethics [61, 62]
Benevolence and nonmaleficence	Ensure AI safety, accuracy, and efficacy Empower human beings Prevent or minimize AI*'*s mental or physical harm to individuals and groups	Benefit patient welfare Improve access to quality and interruption‐free health care Do not cause pain or suffering	Gear effectiveness and efficiency toward reducing morbidity and mortality Create least infringement and least necessity of public justification Use noncoerciveness
Autonomy: ‘Respect people*'*s decision‐making’	Ensure meaningful human oversight Protect privacy and confidentiality Avoid manipulation and Experimentation without informed consent	Respect human self‐determination and decision‐making at individual and relational (communal attachments) levels Provide good professional*–*patient relationship (clinicians*'* confidentiality) and fidelity	Ensure connectedness and solidarity, liberty and self‐ determination
Justice and fairness: ‘Treat people fairly’	Ensure inclusiveness, absence of bias, and equity in AI systems*'* access, use, design, data, and outcomes	Treat persons fairly, equitably, and appropriately: employ distributive justice and socio‐relational justice Provide fair and balanced access	Ensure participation of public and the affected parties: procedural justice Distribute burdens and benefits fairly
Explicability	For technology robustness, ensure AI transparency, resilience, security, accuracy, reliability, and reproducibility Ensure AI intelligibility	Tell the truth	Disclose information and speak truthfully: transparency Avoid paternalism
Responsibility and accountability	Ensure human warranty and third‐ party regulatory bodies*'* approval Implement harm redress mechanisms Ensure responsibility and liability		Ensure communal responsibility and community cohesiveness Be responsible to minimize burden on populations
Sustainability and responsiveness	Ensure AI promotes health and environmental and work sustainability Ensure health protection and promotion		Ensure sustainability of the global environment and human systems: precautionary principle Ensure cost effectiveness and usefulness

*Note:* Adapted from Tiribelli et al. [53].

Specific ethical guidelines for the use of ML in population and public health remain lacking, despite ML's potential to improve public health outcomes: biased outputs can arise not only from data quality and representativeness, already discussed above, but from who is engaged in directing the analysis and how findings are interpreted [53]. Closing this gap will require piloting equity‐focused guidelines across diverse public health contexts, including low‐ and middle‐income countries, with stakeholder engagement between model developers and population health professionals as a core evaluation criterion, alongside ongoing refinement as these guidelines are tested in practice [63].

Recently, a position paper by the Italian Society of Internal Medicine (SIMI) [64] identified AI‐enabled telemedicine as one of the most relevant emerging applications of AI in clinical practice, emphasizing its potential to improve continuity of care, patient monitoring and healthcare accessibility while simultaneously introducing important ethical, legal and organizational challenges. Rather than representing a separate application domain, AI‐based telemedicine exemplifies the broader ethical issues associated with the implementation of AI in healthcare, including data privacy and cybersecurity, algorithmic bias, transparency and explainability of AI‐driven recommendations, clinician accountability and equitable access to digital health services. Furthermore, the increasing integration of AI into remote care highlights the need to preserve the clinician–patient relationship and ensure meaningful human oversight of automated decision‐support systems. Consequently, AI‐enabled telemedicine provides a representative real‐world scenario in which the ethical principles discussed throughout this manuscript can be critically examined, reinforcing the need for robust governance frameworks to ensure the safe, equitable and responsible deployment of AI across healthcare settings.

AI‐based telemedicine became indispensable during COVID‐19 and is expected to play an even greater preventive and continuity‐of‐care role in future pandemics [65, 66, 67]; however, its rapid deployment exposes a set of ethical, legal and socio‐technical challenges that remain insufficiently resolved [53]. Digital care must ensure substantial equivalence with traditional encounters, not only regarding informed consent and clinical safety, but also in safeguarding autonomy and shared decision‐making when AI systems shape medical recommendations in opaque ways. Moreover, the accelerated adoption of remote and data‐driven services has outpaced regulations for accreditation, liability, cross‐jurisdictional practice and data protection, raising concerns around improper data sharing, secondary use of sensitive information, algorithmic bias and inequities in access [68]. Evidence on the clinical effectiveness of video and AI‐supported consultations is promising but still limited, demanding continuous evaluation of quality, fairness and patient trust before such systems can be regarded as substitutes for face‐to‐face care [69, 70, 71]. Crucially, telehealth's reshaping of provider–patient relationships, medical adherence and domestic spaces reveals how emerging technologies transform the very norms of medicine and extend into non‐medical domains, offering three lessons for future AI adoption: measurable benefits may conceal subtle threats to identity and autonomy; impacts vary across populations and specialties, requiring context‐sensitive design; and technologies redefine who clinicians and patients become [72, 73, 74, 75].

### 5.2 ALTAI and Operational Recommendations

Turning these principles into practice requires operational steps: key recommendations for prioritizing ethical issues in AI and ML are summarized in Table 2.

**TABLE 2:** Key recommendations to prioritize ethical issues in the field of AI and ML.

Prioritize partnerships and multistakeholder engagement throughout the total product life cycle.
2 Use ML for dynamic situations, such as public health emergencies, while adhering to ethical standards.
3 Conduct risk assessments and bias mitigation strategies aligned with identified risks
4 Ensure technical transparency by implementing measurement tools for evaluation, monitoring and reproducibility by publicly sharing data sources and methodologies
Ensure technical transparency through explainability: Implement XAI methods (e.g., SHAP, LIME, attention visualization) appropriate to the clinical context; prioritize inherently interpretable models where feasible. Report and evaluate explanation effectiveness rigorously.
ii Conduct counterfactual fairness audits: Use counterfactual reasoning to identify and mitigate demographic disparities in model recommendations. Document audit trails to support regulatory compliance.
5 Foster multidisciplinary dialogue to discuss the potential harms of ML‐related bias and raise awareness among the public and public health community.
6 Creation of a nationally representative data infrastructure and learning environment to support the generation of generalizable knowledge about health effects of AI tools across different settings.
7 Select interventions to support communities considered structurally disadvantaged;
8 Identify incentive structures with the help of market forces and policies to drive changes in the area of health care delivery‐AI.
9 Establish clinician–AI interface standards: Design interfaces that present AI explanations—visual, textual, or counterfactual—in formats validated through human‐centered research with target user groups.

*Note:* Adapted from Pinto et al. [63] and Angus et al. [2].

**FIGURE 1:**
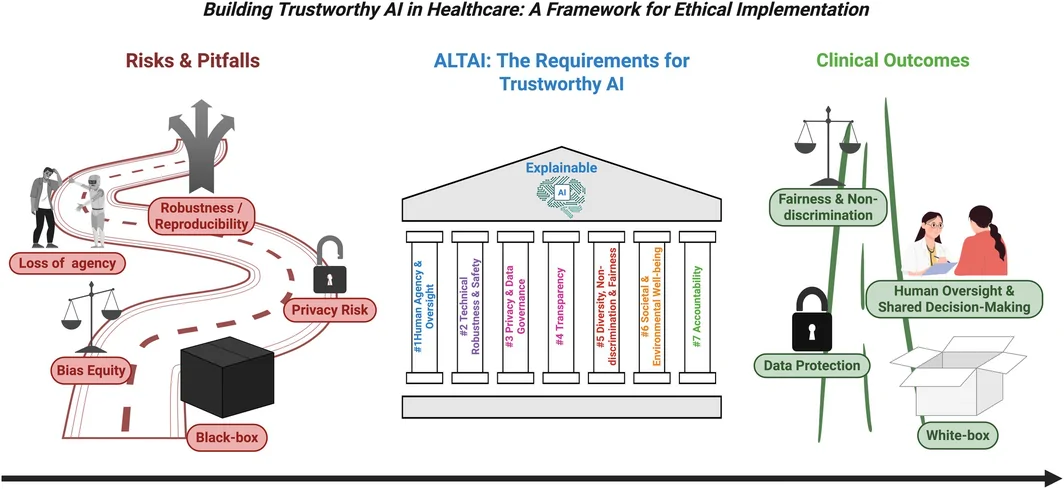
A pathway to trustworthy artificial intelligence (AI) in healthcare through the Assessment List for Trustworthy AI (ALTAI) framework. This conceptual illustration outlines the transition from inherent ethical risks in AI deployment (left: Black‐box models, bias, loss of human agency) to desirable clinical outcomes (right: Fairness, human oversight, data protection). The central pillar represents the operationalization of ethics via the seven requirements of the ALTAI, which serves as a foundational framework to mitigate risks, ensure regulatory compliance and foster the development of transparent, equitable and accountable AI systems for clinical and public health.

While high‐level principles and guidelines set the direction, AI‐based telemedicine, clinical and public‐health projects increasingly require a verifiable mechanism to demonstrate day‐to‐day adherence. In the European context, the Assessment List for Trustworthy AI (ALTAI), a structured self‐assessment developed by the European Commission's High‐Level Expert Group on AI through a multi‐stakeholder piloting phase in 2019, operationalizes these ethical and regulatory commitments by translating them into concrete, auditable checks. This conceptual pathway, guiding the transition from inherent AI risks to trustworthy clinical outcomes via the ALTAI framework, is illustrated in Figure 1.

ALTAI organizes evaluation around seven requirements: Human Agency & Oversight; Technical Robustness & Safety; Privacy & Data Governance; Transparency; Diversity, Non‐discrimination & Fairness; Societal & Environmental Well‐being; Accountability [76, 77, 78].

ALTAI is particularly suitable for clinical and public‐health AI for two reasons. First, it is grounded in fundamental rights and explicitly recommends conducting a Fundamental Rights Impact Assessment (FRIA) prior to the technical self‐assessment, ensuring that equity and patient rights are considered from the outset. Second, it translates high‐level principles into concrete checks. For example, Transparency is decomposed into traceability, explainability and explicit communication of system limitations, aligning with the manuscript's emphasis on XAI and meaningful human oversight. ALTAI also clarifies the content of Accountability—including auditability, risk management and redress mechanisms—and links these to practical enablers such as traceability and logging from early design to support audits over the system lifecycle. Likewise, the Fairness requirement operationalizes bias mitigation through ongoing monitoring, subgroup analyses and stakeholder participation to define context‐appropriate fairness metrics. Finally, ALTAI extends beyond model accuracy to Technical Robustness & Safety (e.g., distribution shift, adversarial risk, fall‐back plans, reproducibility) and to Societal/Environmental well‐being, including energy use and carbon footprint—considerations increasingly expected by funders and regulators. Accordingly, ALTAI can be incorporated as a living checklist across the total product life cycle—requirements capture, data governance, model development, deployment, monitoring and periodic reassessment. A mapping of common clinical‐AI challenges to ALTAI requirements, with suggested evidence artefacts for documentation, is provided in Table 3.

**TABLE 3:** Mapping common ethical/operational challenges in clinical & public‐health AI to ALTAI requirements and concrete evidence artefacts.

Ethical/Operational challenge	Relevant ALTAI requirement(s)	What to demonstrate	Example evidence/artefacts
Preserving human oversight and contestability	#1 Human Agency & Oversight	Clear override pathways, calibrated confidence communication, and predefined low‐confidence fallbacks	User interface (UI) affordances to overrule; calibrated confidence/uncertainty displays; escalation policies; training materials for end users.
Robustness under distribution shift; safety in high‐risk use	#2 Technical Robustness & Safety	Adversarial/misuse‐case analysis; reliability under distribution shift; predefined fail‐safe plans; reproducibility	Red‐team exercises and penetration‐testing reports; stress/reliability testing; predefined fail‐safe rules and fallback clinical workflows; version‐controlled, reproducible data/model pipelines.
Privacy, data minimisation, and lawful processing	#3 Privacy & Data Governance	Data Protection Impact Assessment (DPIA) where required; governance processes; appropriate de‐identification	DPIA record; Data Protection Officer (DPO) involvement; encryption/pseudonymisation/anonymisation procedures; data‐governance standard operating procedures (SOPs).
Making model behaviour intelligible to clinicians	#4 Transparency (traceability, explainability, communication)	Ability to trace inputs, models, and outputs; provide user‐level explanations; communicate limitations and intended use	Data/model lineage logs; per‐case explanations; user‐facing model card (intended use, limitations).
Detecting and mitigating bias; defining fairness with stakeholders	#5 Diversity, Non‐discrimination & Fairness	Strategy to avoid unfair bias; subgroup metrics; participatory definition of fairness	Bias‐monitoring plan; subgroup performance dashboards; records of community/stakeholder consultation.
Environmental and societal externalities	#6 Societal & Environmental Well‐being	Energy/Carbon accounting and reduction measures; assessment of broader societal and workforce impacts	Training/inference energy logs; carbon estimates; mitigation plan; assessment of impact on work/skills.
Auditability, risk management, and redress	#7 Accountability	Audit trail; internal/external audit readiness; redress by design	End‐to‐end logs; risk register; incident‐response SOPs; user‐facing redress pathways.

*Note:* Adapted from the Assessment List for Trustworthy Artificial Intelligence (ALTAI), developed by the European Commission's High‐Level Expert Group on Artificial Intelligence (2020) [77].

## 6 Conclusions and Perspectives

AI tools, including network medicine and telemedicine, are increasingly integrated into public health systems worldwide, extending the potential benefits discussed throughout this review, from diagnostics to drug discovery, toward personalized treatment strategies grounded in a patient's genotype or sequence variants rather than population‐level averages, a further step toward precision medicine. Additional benefits include optimized disease prevention strategies such as behavioural modification and pharmaco‐prevention, and reduced exposure to potentially toxic medications. The implementation of AI in the field of clinical health care, however, also comes with ethical risks and this issue must be detected, prevented or mitigated for the responsible use of AI‐based telemedicine in public health. Such an urgent need will inevitably intersect all stakeholders, including industries, clinicians, patients and society as well. Notably, in the absence of a context sensitive ethical framework to closely assess AI technology and detect potential ethical risks, AI‐based telemedicine for public health will be jeopardized, undermining people's trust in AI‐based tools. Any effort should be undertaken to avoid that public health actors will become reluctant to implement, experience and ultimately adopt AI‐related tools to improve public health globally. As this review has outlined, translating these benefits into practice depends as much on data quality, paradigm‐appropriate transparency and operational frameworks such as ALTAI, as on technological potential itself.

## Author Contributions

P.P., S.T., conceptualization. M.K., P.N., writing original draft. P.P., S.T., writing and editing.

## Funding

This research was supported by the P.N. RIC 2021–2027 Call D.D 307/2025, Action 1.1.2, Title project ‘Omics and Medicine for New Intelligent Applications – Biomedical Research and Innovation from THE to Develop Growth and Excellence in the South’ – MI 0002309—Project ID QIIR112_00059, CUP B99H26000500007, co‐funded by the European Union – European Regional Development Fund (ERDF/FESR). The authors acknowledge the project On Foods: P.P. is a grant recipient and M.K. is an assistant researcher (RTDA) in a project funded under the National Recovery and Resilience Plan (NRRP), Mission 4 Component 2 Investment 1.3—Call for tender No. 341 of 15/03/2022 of Italian Ministry of University and Research funded by the European Union—Next Generation EU Award Number: Project code PE0000003, Concession Decree No. 1550 of 11/10/2022 adopted by the Italian Ministry of University and Research, CUP D93C22000890001, Project title ‘Research and innovation network on food and nutrition Sustainability, Safety and Security—Working ON Foods’ (ONFoods). PRIMA project: P.P. is the coordinator of the B4HT project ‘Box for Health by Tradition & Innovation: promoting sustainable mediterranean diet by Healthy Foods’ funded by the PRIMA project, Section 2—Multi‐topic 2022. Project partners: University of Bari Aldo Moro (Italy), University of Genoa (Italy), Lebanese University (Lebanon) and University of Monastir (Tunisia). PAS GRAS project: P.P. is the recipient of HORIZON‐HLTH‐2022‐STAYHLTH‐01‐05‐two‐stage Project 101080329—PAS GRAS.

## Ethics Statement

The authors have nothing to report.

## Conflicts of Interest

The authors declare no conflicts of interest.

## Data Availability

The authors have nothing to report.
